# Supplementary figures and images for: Positive effect of an electrolyzed reduced water on gut permeability, fecal microbiota and liver in an animal model of Parkinson’s disease
Source: PLoS One. 2019 Oct 10;14(10):e0223238. doi: 10.1371/journal.pone.0223238 (PMC6786615; doi:10.1371/journal.pone.0223238)

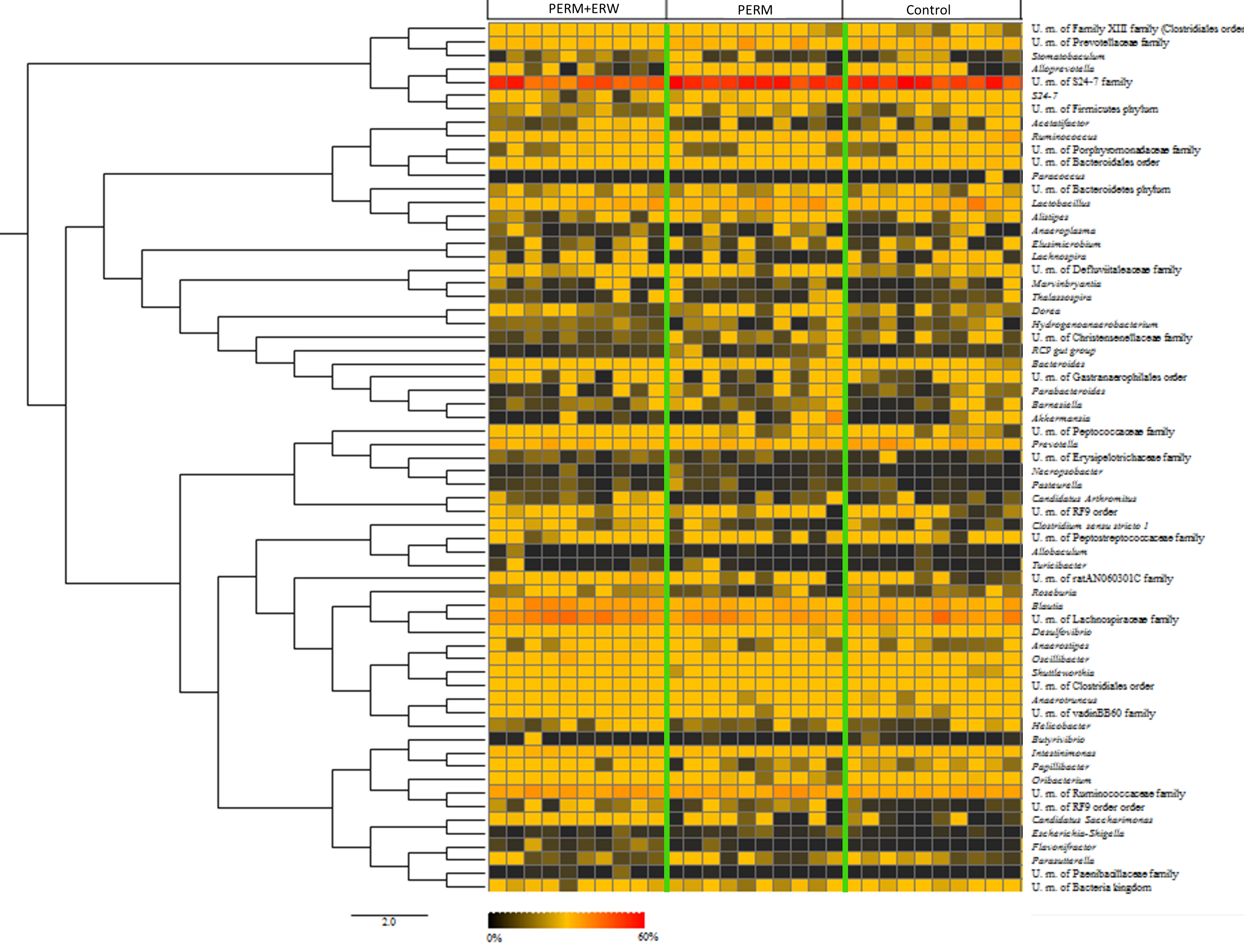

Supplement: S1 Fig — The heat map shows the relative abundance of bacterial genera identified in each sample, the three groups (n = 10 rats per group) are separated by green lines. Hierarchical clustering was performed using Pearson correlation. (TIF) [file pone.0223238.s001.tif]
