# Supplementary material for: Positive effect of an electrolyzed reduced water on gut permeability, fecal microbiota and liver in an animal model of Parkinson’s disease
Source: PLoS One. 2019 Oct 10;14(10):e0223238. doi: 10.1371/journal.pone.0223238 (PMC6786615; doi:10.1371/journal.pone.0223238)
Supplement: S1 Table — Relative abundance of bacterial genera in fecal microbiota of 2-months-old rats treated in early life with vehicle (C), permethrin (PERM) or permethrin+electrolyzed reduced water (PERM+ERW). All data are expressed as means ± SEM. Group sizes: control (n = 10), PERM (n = 10) and PERM+ERW (n = 10). Significance was determined using Kruskal-Wallis (a) or ANOVA (b). *P<0.05; **P<0.01; ***P<0.001. (PDF) [file pone.0223238.s002.pdf]

Taxa	CONTROL mean	CONTROL SD	PERM mean	PERM SD	PERM+ERW mean	PERM+ERW SD	P-value	C *vs* PERM	C *vs* PERM+ERW	PERM *vs* PERM+ERW
**Phylum**										
Bacteroidetes [b]	57.43	11.56	58.41	7.68	43.06	12.13	**0.004**		**	**
Candidate division TM7 [a]	0.11	0.29	0.08	0.10	0.16	0.07	**0.014**		*	
Cyanobacteria [a]	0.10	0.09	0.15	0.31	0.12	0.14	0.748			
Elusimicrobia [a]	0.10	0.14	0.06	0.06	0.04	0.05	0.625			
Firmicutes [b]	40.89	12.22	38.34	8.03	55.33	11.85	**0.003**		**	**
Proteobacteria [a]	0.38	0.18	0.43	0.18	0.60	0.24	**0.042**		*	
Tenericutes [a]	0.70	1.51	0.49	0.69	0.45	0.34	0.467			
Verrucomicrobia [a]	0.15	0.25	1.91	5.26	0.10	0.31	0.591			
U.m. of bacteria kingdom [b]	0.11	0.03	0.07	0.02	0.08	0.02	**0.017**	*	*	
Others [a]	0.03	0.01	0.04	0.01	0.04	0.02	0.322			
**Family**										
Bacteroidaceae [a]	0.21	0.24	0.34	0.42	0.16	0.08	0.761			
Porphyromonadaceae [a]	0.10	0.09	0.46	0.87	0.10	0.08	0.051			
Prevotellaceae [b]	9.68	4.30	8.81	4.48	6.23	3.66	0.176			
ratAN060301C [a]	0.44	0.62	0.16	0.16	1.80	2.20	**0.029**		*	**
Rikenellaceae [a]	0.18	0.22	0.21	0.12	0.08	0.08	0.067			
S24-7 [b]	45.47	10.32	47.66	7.55	34.05	11.23	**0.009**		*	*
U.m. of Bacteroidales order [a]	1.26	0.99	0.70	0.26	0.55	0.29	**0.050**		*	
U.m. of Candidate division TM7 [a]	0.11	0.29	0.08	0.10	0.16	0.07	**0.014**		*	
U.m. of Gastranaerophilales order [a]	0.10	0.09	0.15	0.31	0.12	0.14	0.748			
Elusimicrobiaceae [a]	0.10	0.14	0.06	0.06	0.04	0.05	0.625			
Lactobacillaceae [a]	5.93	5.95	6.02	4.63	4.51	3.79	0.776			
Christensenellaceae [a]	0.07	0.06	0.16	0.19	0.03	0.01	**0.005**			**
Clostridiaceae 1 [a]	0.05	0.04	0.05	0.04	0.17	0.10	**0.005**		*	**
Defluviitaleaceae [a]	0.07	0.02	0.14	0.08	0.13	0.08	**0.036**	*		
Family XIII clostridiales order [b]	0.11	0.06	0.14	0.07	0.15	0.02	0.141			
Lachnospiraceae [b]	20.88	9.87	17.92	6.65	31.23	10.09	**0.006**		*	**
Peptococcaceae [b]	0.12	0.08	0.10	0.06	0.15	0.05	0.297			
Peptostreptococcaceae [a]	0.21	0.15	0.22	0.21	0.29	0.34	0.866			
Ruminococcaceae [a]	12.97	3.97	13.10	3.58	17.99	4.19	**0.011**		*	
vadinBB60 [a]	0.14	0.06	0.17	0.15	0.23	0.11	0.117			
U.m. of clostridiales order [b]	0.21	0.09	0.21	0.08	0.34	0.09	**0.002**		**	**
Erysipelotrichaceae [a]	0.05	0.07	0.05	0.05	0.07	0.05	0.159			
Rhodospirillaceae [a]	0.03	0.05	0.06	0.10	0.04	0.06	0.971			
Alcaligenaceae [a]	0.07	0.06	0.08	0.07	0.07	0.08	0.827			
Desulfovibrionaceae [a]	0.18	0.08	0.19	0.08	0.33	0.15	**0.014**		*	*
Helicobacteraceae [a]	0.05	0.04	0.05	0.04	0.13	0.15	0.162			

Enterobacteriaceae [a]	0.01	0.01	0.02	0.01	0.01	0.01	0.061			
Pasteurellaceae [a]	0.01	0.02	0.03	0.04	0.02	0.02	0.254			
Anaeroplasmataceae [a]	0.04	0.10	0.03	0.05	0.03	0.04	0.623			
U. m. of RF9 order [a]	0.65	1.53	0.44	0.65	0.40	0.34	0.376			
Paenibacillaceae [a]	0.00	0.00	0.00	0.00	0.01	0.01	0.166			
Verrucomicrobiaceae [a]	0.15	0.25	1.91	5.26	0.10	0.31	0.591			
U. m. of Bacteria kingdom [b]	0.11	0.03	0.07	0.02	0.08	0.02	**0.017**		*	*
Others [b]	0.23	0.14	0.20	0.05	0.19	0.05	0.946			
**Genus**										
Bacteroides [a]	0.21	0.24	0.34	0.42	0.16	0.08	0.761			
Barnesiella [a]	0.04	0.01	0.13	0.08	0.04	0.01	0.317			
Parabacteroides [a]	0.04	0.02	0.30	0.20	0.05	0.02	0.150			
U.m. of porphyromonadaceae family [a]	0.43	0.15	0.11	0.03	0.13	0.03	0.217			
Alloprevotella [a]	0.57	0.18	0.67	0.22	0.16	0.08	0.065			
Prevotella [a]	6.22	1.31	3.57	0.75	3.92	0.89	0.199			
U. m. of Prevotellaceae family [a]	2.88	0.68	4.57	1.48	2.15	0.52	0.671			
U.m. of rat AN 060301C family [a]	0.44	0.20	0.16	0.05	1.80	0.70	**0.002**		*	**
Alistipes [a]	0.17	0.07	0.17	0.03	0.07	0.02	0.091			
S24-7 [b]	0.20	0.04	0.20	0.03	0.10	0.02	**0.035**			*
U. m. of S24-7 family [b]	44.84	3.23	47.35	2.38	33.82	3.53	**0.010**		*	*
U.m. of Bacteroidales order [a]	1.26	0.31	0.70	0.08	0.55	0.09	**0.050**		*	
Candidatus Saccharimonas [a]	0.11	0.09	0.08	0.03	0.16	0.02	**0.014**		*	
U.m. of Gastranaerophilales order [a]	0.10	0.03	0.15	0.10	0.12	0.04	0.748			
Elusimicrobium [a]	0.10	0.04	0.06	0.02	0.04	0.01	0.625			
Lactobacillus [a]	5.93	1.88	6.02	1.46	4.51	1.20	0.776			
U. m. of Christensenellaceae family [a]	0.06	0.02	0.14	0.06	0.03	0.00	**0.041**			*
Clostridium sensu stricto 1[a]	0.03	0.01	0.02	0.01	0.12	0.03	**0.003**		*	**
U. m. of Defluviitaleaceae family [a]	0.07	0.01	0.14	0.03	0.13	0.02	**0.036**	*		
U.m.of familyXIII Clostridiales order [b]	0.09	0.01	0.13	0.02	0.12	0.01	0.171			
Acetatifactor [a]	0.07	0.02	0.04	0.03	0.07	0.02	0.053			
Anaerostipes [a]	0.14	0.06	0.16	0.06	0.45	0.22	0.196			
Blautia[b]	5.78	0.99	5.07	0.87	10.71	1.50	**0.003**		**	**
Butyrivibrio [a]	0.01	0.00	0.00	0.00	0.02	0.02	0.349			
Dorea [a]	0.05	0.01	0.13	0.03	0.07	0.02	0.055			
U.m. of Lachnospiraceae family [b]	13.29	2.00	11.85	1.39	18.37	1.73	**0.031**		*	*
Lachnospira [a]	1.05	0.41	0.02	0.01	0.47	0.24	**0.024**	*		
Marvinbryantia [a]	0.03	0.02	0.14	0.12	0.13	0.10	0.419			

Oribacterium [a]	0.13	0.01	0.21	0.08	0.30	0.05	0.051			
Roseburia [a]	0.05	0.01	0.03	0.01	0.10	0.03	**0.012**			**
Shuttleworthia [a]	0.23	0.05	0.17	0.03	0.44	0.09	**0.014**			*
Stomatobaculum [a]	0.03	0.01	0.05	0.02	0.06	0.02	0.486			
U. m. of Peptococcaceae family[b]	0.11	0.03	0.09	0.02	0.14	0.01	0.300			
U.m.of Peptostreptococcaceae family [a]	0.21	0.05	0.22	0.07	0.29	0.11	0.866			
Anaerotruncus [a]	0.16	0.03	0.18	0.04	0.29	0.04	**0.038**		***	**
Flavonifractor [a]	0.00	0.00	0.00	0.00	0.02	0.01	**0.002**		*	
U.m. of Ruminococcaceae family [b]	7.80	0.55	9.20	1.03	10.86	0.73	**0.037**		*	*
Intestinimonas [a]	1.81	0.17	1.81	0.19	3.20	0.38	**0.004**			*
Oscillibacter [a]	0.69	0.10	0.52	0.15	1.66	0.58	**0.005**			**
Papillibacter [a]	0.07	0.01	0.07	0.02	0.18	0.03	**0.005**		*	**
Ruminococcus [a]	2.37	0.90	1.26	0.48	1.70	0.41	0.283			
U. m. of vadin BB60 family [a]	0.14	0.02	0.17	0.05	0.23	0.04	0.117			
U. m. of Clostridiales order [b]	0.21	0.03	0.21	0.03	0.34	0.03	**0.002**		**	**
U.m. of Erysipelotrichaceae family [a]	0.03	0.02	0.02	0.00	0.03	0.00	0.101			
Turicibacter [a]	0.00	0.00	0.02	0.01	0.02	0.01	0.944			
Thalassospira [a]	0.03	0.01	0.06	0.03	0.04	0.02	0.928			
Parasutterella [a]	0.07	0.02	0.08	0.02	0.07	0.03	0.827			
Desulfovibrio [a]	0.18	0.03	0.19	0.02	0.33	0.05	**0.014**		*	*
Helicobacter [a]	0.05	0.01	0.05	0.01	0.13	0.05	0.162			
Escherichia-Shigella [a]	0.01	0.00	0.02	0.00	0.01	0.00	0.106			
Anaeroplasma [a]	0.04	0.03	0.03	0.01	0.03	0.01	0.623			
U. m. of RF9 order [a]	0.64	0.48	0.39	0.19	0.32	0.10	0.722			
U. m. of RF9 order order [a]	0.01	0.00	0.05	0.02	0.09	0.03	**0.007**		**	
U. m. of Paenibacillaceae family [a]	0.00	0.00	0.00	0.00	0.01	0.00	0.166			
Akkermansia [a]	0.15	0.08	1.91	1.66	0.10	0.10	0.591			
U. m. of Bacteria kingdom[b]	0.11	0.01	0.07	0.01	0.08	0.01	**0.017**	*	*	
Others [a]	0.44	0.05	0.48	0.03	0.48	0.04	0.204			
